# Prediction of Blood Lipid Phenotypes Using Obesity-Related Genetic Polymorphisms and Lifestyle Data in Subjects with Excessive Body Weight

**DOI:** 10.1155/2018/4283078

**Published:** 2018-11-19

**Authors:** Omar Ramos-Lopez, Jose I. Riezu-Boj, Fermin I. Milagro, Marta Cuervo, Leticia Goni, J. A. Martinez

**Affiliations:** ^1^Department of Nutrition, Food Science and Physiology, and Center for Nutrition Research, University of Navarra, Pamplona, Spain; ^2^Navarra Institute for Health Research (IdiSNA), Pamplona, Spain; ^3^CIBERobn, Fisiopatología de la Obesidad y la Nutrición, Carlos III Health Institute, Madrid, Spain; ^4^Madrid Institute of Advanced Studies (IMDEA Food), Madrid, Spain

## Abstract

**Background and Aim:**

Individual lipid phenotypes including circulating total cholesterol (TC), low-density lipoprotein cholesterol (LDL-c), high-density lipoprotein cholesterol (HDL-c), and triglycerides (TG) determinations are influenced by gene-environment interactions. The aim of this study was to predict blood lipid level (TC, LDL-c, HDL-c, and TG) variability using genetic and lifestyle data in subjects with excessive body weight-for-height.

**Methods:**

This cross-sectional study enrolled 304 unrelated overweight/obese adults of self-reported European ancestry. A total of 95 single nucleotide polymorphisms (SNPs) related to obesity and weight loss were analyzed by a targeted next-generation sequencing system. Relevant genotypes of each SNP were coded as 0 (nonrisk) and 1 (risk). Four genetic risk scores (GRS) for each lipid phenotype were calculated by adding the risk genotypes. Information concerning lifestyle (diet, physical activity, alcohol drinking, and smoking) was obtained using validated questionnaires. Total body fat (TFAT) and visceral fat (VFAT) were determined by dual-energy X-ray absorptiometry.

**Results:**

Overall, 45 obesity-related genetic variants were associated with some of the studied blood lipids. In addition to conventional factors (age, sex, dietary intakes, and alcohol consumption), the calculated GRS significantly contributed to explain their corresponding plasma lipid trait. Thus, HDL-c, TG, TC, and LDL-c serum concentrations were predicted by approximately 28% (optimism-corrected adj. *R* ^2^ = 0.28), 25% (optimism-corrected adj. *R* ^2^ = 0.25), 24% (optimism-corrected adj. *R* ^2^ = 0.24), and 21% (optimism-corrected adj. *R* ^2^=0.21), respectively. Interestingly, GRS were the greatest contributors to TC (squared partial correlation (PC^2^) = 0.18) and LDL-c (PC^2^ = 0.18) features. Likewise, VFAT and GRS had a higher impact on HDL-c (PC^2^ = 0.09 and PC^2^ = 0.06, respectively) and TG levels (PC^2^ = 0.20 and PC^2^ = 0.07, respectively) than the rest of variables.

**Conclusions:**

Besides known lifestyle influences, some obesity-related genetic variants could help to predict blood lipid phenotypes.

## 1. Introduction

Triglycerides, cholesterol, and related lipoproteins are major constituents of the lipid fraction of the human body, playing essential physiological roles such as cell membrane stability, energy storage, hormone and bile acid syntheses, dietary fat absorption and assembling, stress response, cell signaling, and calcium metabolism [1, 2]. However, abnormalities in lipid metabolism may lead to the onset and development of several metabolic disorders, including cardiovascular disease features [3]. In this context, elevated plasma levels of total cholesterol (TC), low-density lipoprotein cholesterol (LDL-c), and triglycerides (TG) have been associated with the risk of coronary heart disease, whereas high concentrations of high-density lipoprotein cholesterol (HDL-c) may exert a protective effect [4].

Growing scientific evidence suggests that gene-environment interactions may influence plasma lipid phenotypes [5]. Lifestyle factors such as diet, physical activity, alcohol drinking, and smoking have been recognized as important determinants of the blood lipid profile [5]. Moreover, genome-wide association studies (GWAS) and gene-candidate analyses have identified a number of common genetic variants associated with diverse lipid traits [6]. Also, specific genetic risk scores (GRS) including multiple gene loci have accounted for dyslipidemia susceptibilities and predisposition to related health risks in some populations [7, 8].

Furthermore, differences in cholesterol and TG outcomes according to genotypes of single nucleotide polymorphisms (SNPs) in response to dietary interventions have been reported [9–11]. Nevertheless, most available studies mainly include SNPs in genes directly implicated in lipid metabolism (uptake, transport, and signaling) [12, 13], whereas those related to body weight regulation and obesity remain less explored. Together, these insights reveal a genetic component implicated in lipid homeostasis that may partially explain the variability in circulating lipids among individuals. In addition, this knowledge can help to specifically establish personalized nutritional guidelines that complement the general recommendations to the prevention and precision management of dyslipidemia [14, 15]. Hence, the aim of this research was to predict blood lipid profiles using genetic and environmental data in subjects with excessive body weight-for-height.

## 2. Materials and Methods

### 2.1. Subjects

This cross-sectional study enrolled 304 unrelated (nonconsanguineous) Spanish adults of self-reported European ancestry, who presented overweight (BMI: 25–29.9 kg/m^2^) and obesity (BMI: 30–40 kg/m^2^). Subjects were recruited at the Center for Nutrition Research of the University of Navarra in the city of Pamplona, Navarra, Spain. Major exclusion criteria included a history of diabetes mellitus, cardiovascular disease and hypertension, pregnant or lactating women, and current use of lipid-lowering drugs. Patients with diagnosed primary hyperlipidemia were also excluded. This investigation followed the ethical principles for medical research in humans from the 2013 Helsinki Declaration [16]. Moreover, the research protocol was properly approved by the Research Ethics Committee of the University of Navarra (ref. 132/2015). A written informed consent from each participant was obtained before the inclusion in the study.

### 2.2. Anthropometry and Blood Pressure

Anthropometric measurements such as height (cm), body weight (kg), and waist circumference (WC, cm) were collected at the fasting state by trained nutritionists following validated procedures [17]. BMI was calculated as the ratio between weight and squared height (kg/m^2^). Total body fat (TFAT, kg) and visceral fat (VF, kg) were quantified by dual-energy X-ray absorptiometry according to instructions provided by the supplier (Lunar Prodigy, software version 6.0, Madison, WI, USA). Systolic blood pressure (SBP, mmHg) and diastolic blood pressure (DBP, mmHg) were measured with an automated sphygmomanometer according to standardized criteria as described by the World Health Organization and the International Society of Hypertension [18].

### 2.3. Biochemical Measurements

Blood samples were drawn by venipuncture after an overnight fast. Biochemical measurements including glucose (mg/dl), total cholesterol (TC, mg/dl), high-density lipoprotein cholesterol (HDL-c, mg/dl), and triglycerides (TG, mg/dl) were determined in an automatic analyzer (Pentra C200, HORIBA Medical) using appropriate kits provided by the company. Low-density lipoprotein cholesterol (LDL-c, mg/dl) was calculated with the Friedewald formula [19]. The following cutoffs for the Spanish population were used to the diagnosis of dyslipidemia: hypercholesterolemia (TC ≥ 200 mg/dl), high LDL-c (LDL-c ≥ 130 mg/dl), hypoalphalipoproteinemia (HDL-c < 40 mg/dl in men and < 50 mg/dl in women), and hypertriglyceridemia (TG ≥ 150 mg/dl), as reported elsewhere [20].

### 2.4. Lifestyle Factors

A validated semiquantitative food frequency questionnaire was used to evaluate habitual consumption (daily, weekly, monthly, or never) of 137 foods during the previous year [21]. Energy and nutrient intakes were further calculated with an ad hoc computer program based on the standard Spanish food composition tables [22].

The physical activity level was estimated using a validated questionnaire [23]. The volume of activity was expressed in metabolic equivalents (METs), as described elsewhere [24].

Current smoking and drinking habits were evaluated through valid medical questionnaires. Alcohol consumption higher than 40 g of ethanol/d in men and 20 g of ethanol/d in women was considered clinically significant [25].

### 2.5. SNP Selection and Genotyping

A total of 95 genetic variants related to obesity and weight loss as well as interactions with dietary prescriptions were analyzed after an exhaustive bibliographic review following PRISMA criteria [14, 15, 26, 27], whose genomic characteristics are presented (Supplementary Table 1).

Buccal samples were collected with a liquid-based kit (ORAcollect-DNA, OCR-100, DNA Genotek Inc., Ottawa, Canada). Subsequently, genomic DNA was isolated using the Maxwell® 16 Buccal Swab LEV DNA Purification Kit in the Maxwell® 16 Instrument (Promega Corp., Madison, WI, USA) according to the manufacturer's protocol. A customized panel of primers to amplify the regions containing the selected SNPs was designed using the “online” application of Thermo Fisher AmpliSeq Designer (https://www.ampliseq.com). Overall, the amplicon average size was 185 bp. The amplicon library for massive sequencing was constructed with the custom-designed panel and the Ion AmpliSeq™ Library Kit 2.0 (Thermo Fisher Scientific Inc., Waltham, MA, USA) according to the manufacturer's protocol.

Genotyping was performed by targeted next-generation sequencing in the Ion Torrent PGM™ equipment (Thermo Fisher Scientific Inc., Waltham, MA, USA), as described elsewhere [9, 10]. Raw data were processed in the Ion Torrent Suite™ Server version 5.0.4 (Thermo Fisher Scientific Inc., Waltham, MA, USA) using the *Homo sapiens* (HG19) as the reference genome for the alignment. A custom-designed Bed file was used to locate the SNPs of interest. Genetic variants were identified with the Torrent Variant Caller 5.0 (Thermo Fisher Scientific Inc., Waltham, MA, USA) with a minimum coverage value of 25 sequences [28]. Hardy-Weinberg equilibrium (HWE) was estimated with the Convert (version 1.31) and the Arlequin software (version 3.0). Furthermore, the analysis of molecular variance (AMOVA) test using the 95 SNPs was performed in the Arlequin software in order to corroborate the homogeneity of the sample.

### 2.6. GRS Calculation

Once the 95 SNPs were genotyped, four individual GRS were calculated for each lipid trait (TC, LDL-c, HDL-c, and TG) according to the following steps. First, in order to avoid bias and overfitting in the preselection of SNPs [29], ANOVA tests were run to discard those clearly not associated (*P* > 0.25) with some of the four blood lipid phenotypes. The genotypes of the rest of SNPs (*n* = 74) were differentially coded as 0 (nonrisk) and 1 (risk) based on the observed average values of each lipid between the three genotypes using post hoc tests (Bonferroni or Dunnett's T3). A risk genotype was defined as the one that was associated with increased concentrations of fasting TC, LDL-c, and TG or decreased HDL-c levels. Genotypes with similar effects were grouped in a single category. In a third step, Student's *t*-tests were further applied to assess statistical differences between the categorized genotype groups (risk vs. nonrisk). Then SNPs showing at least a marginal statistical trend (*P* < 0.10) were selected (*n* = 54) to design each specific GRS, excluding those with a low prevalence (<10%) in either genotype category (risk and nonrisk) to avoid model instability (*n* = 9). From the remaining 45 SNPs, four different GRS (GRS_TC, GRS_LDL-c, GRS_HDL-c, and GRS_TG) were constructed by adding the risk genotypes of the corresponding SNPs for each study lipid trait (Supplementary Tables 2a–2d). Analyses were performed using the four GRS as continuous and categorical variables.

### 2.7. Statistical Analyses

Continuous variables were expressed as means ± standard deviations, while dichotomous variables were presented as numbers and percentages. Normality of study variables was screened by the Kolmogorov-Smirnov test. All principal variables including TC, LDL-c, HDL-c, and TG were normally distributed (*P* > 0.05).

In addition to genetic variants, other conventional predictors of blood lipid levels were evaluated including age, sex, BMI (kg/m^2^), adiposity markers (TFAT and VFAT), physical activity (METs), total energy (kcal), and macronutrient intakes (% E) as well as smoking and drinking habits. Relevant interactions between genetic and lifestyle factors were calculated with simple linear regression tests. Statistical differences in blood lipids by predictor categories were assessed by Student's *t*-tests.

The prediction of the variability in all blood lipid levels was performed using multiple linear regression models. For this purpose, three statistical approaches were used: least-angle regression (LARS) [30], best subset regression procedure (BSRP) [31], and bootstrapping stepwise method (BSM) [32]. In order to select the most robust model, all candidate predictive models were corrected for optimism and overfitting following Harrell's bootstrapping algorithm [33]. This method is based on using bootstrapped datasets to internally validate the linear regression models as well as to repeatedly quantify the degree of overfitting in the model-building process. Moreover, squared partial correlations (PC^2^) were used to estimate the individual contribution of each predictor to the blood lipid variability.

Statistical analyses were performed in the statistical program STATA 12 (StataCorp LLC, College Station, TX, USA; http://www.stata.com). A Venn diagram was constructed online (http://bioinfogp.cnb.csic.es/tools/venny/) in order to show common and uncommon SNPs associated with each of the studied blood lipids. Figure plots concerning comparisons of blood lipid levels between predictor categories were created using the GraphPad Prism® software, version 6.0C (La Jolla, CA, USA). Statistical significance was based on a *P* value lower than 0.05.

## 3. Results

The anthropometric, biochemical, and nutritional characteristics of the study population are reported (Table 1). Overall, 70% (*n* = 212) of subjects were women. According to the BMI classification criteria of the World Health Organization, 38% of individuals were overweight (*n* = 114), and 62% (*n* = 190) presented obesity. The average values of TC and LDL-c were above the reference limits. The frequencies of hypercholesterolemia, high LDL-c, and low HDL-c (also known as hypoalphalipoproteinemia) were 65% (*n* = 199), 59% (*n* = 179), and 23% (*n* = 69), respectively, whereas 15% of the study population had hypertriglyceridemia (*n* = 45). The nutritional pattern of the study population was characterized by a high consumption of energy derived from fat (40.4%) and a concomitant low intake of carbohydrates (40.7%) with respect to general nutritional recommendations for the Spanish population. The frequencies of smoking and drinking habits were 21.9 and 13.5%, respectively (Table 1).

**Figure 1:**
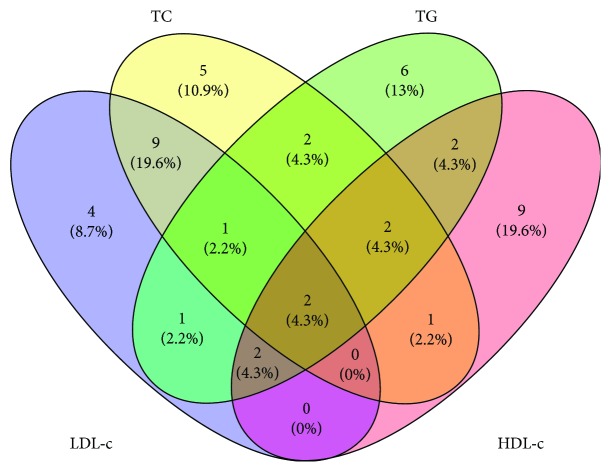
Venn diagram showing the number of SNPs associated with blood lipid levels. TC: total cholesterol; TG: triglycerides; LDL-c: low-density lipoprotein cholesterol; HDL-c: high-density lipoprotein cholesterol.

A total of 45 obesity-related genetic variants were associated with some of the studied blood lipid levels (Supplementary Tables 2a–2d). Of these, 2 SNPs were common among all lipids: rs1685325 (*UCP3*) and rs894160 (*PLIN1*). On the other hand, 22 SNPs were exclusively related to a specific lipid—4 for TC: rs569805 (*ABCB11*), rs494874 (*ABCB11*), rs1801260 (*CLOCK*), and rs6013029 (*CTNNBL1*); 3 for LDL-c: rs7799039 (*LEP*), rs7498665 (*SH2B1*), and rs7359397 (*SH2B1*); 9 for HDL-c: rs2815752 (*NEGR1*), rs2943641 (*IRS1*), rs2419621 (*ACSL5*), rs6265 (*BDNF*), rs11030104 (*BDNF*), rs4769873 (*ALOX5AP*), rs9939609 (*FTO*), rs6567160 (*MC4R*), and rs2287019 (*QPCTL*); and 6 for TG: rs324420 (*FAAH*), rs2959272 (*PPARG*), rs1386835 (*PPARG*), rs709158 (*PPARG*), rs1175540 (*PPARG*), rs1800544 (*ADRA2A*) (Figure 1). The distribution of genotypes of most obesity-predisposing SNPs was concordant with the Hardy-Weinberg equilibrium principle, except for rs1386835 (*PPARG*), rs17782313 (*MC4R*), rs2287019 (*QPCTL*), and rs3813929 (*HTR2C*), as shown in Supplementary Table 1. AMOVA analyses revealed no significant differentiation within the sample (*P* > 0.05).

The performance of the three multiple linear regression models predicting blood lipid profiles are reported (Supplementary Tables 3a–3d). After optimism correction, the best model explaining TC, LDL-c, and HDL-c serum concentrations was then obtained using the BSRP approach, whereas TG levels were better predicted by the BSM method (Table 2). Of note, all models included the calculated GRS in addition to conventional factors such as age, sex, dietary intakes, and alcohol consumption. The highest number of predictors was found for HDL-c, whereas LDL-c was only influenced by the GRS_LDL-c and age. No statistically significant interactions between the 4 GRS and lifestyle variables were found. Overall, HDL-c, TG, TC, and LDL-c variabilities were explained in approximately 28% (optimism-corrected adj. *R* ^2^ = 0.28), 25% (optimism-corrected adj. *R* ^2^ = 0.25), 24% (optimism-corrected adj. *R* ^2^ = 0.24), and 21% (optimism-corrected adj. *R* ^2^ = 0.21), respectively (Table 2).

**Figure 2:**
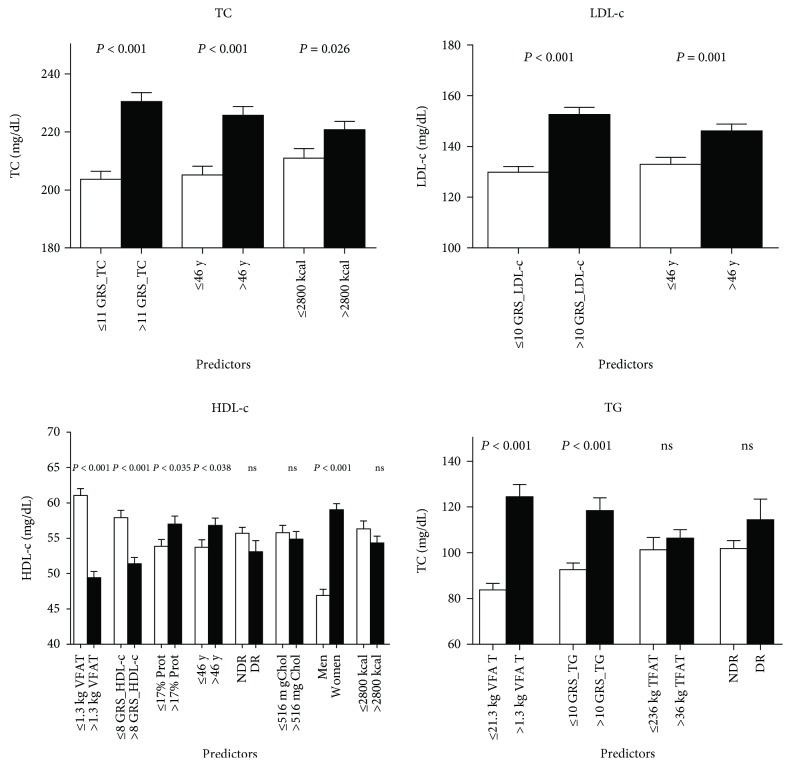
Comparisons of average blood lipids levels between predictor categories based on the median values. Data are expressed as means ± standard errors and sorted in descending order by the effect sizes. TC: total cholesterol; LDL-c: low-density lipoprotein cholesterol; HDL-c: high-density lipoprotein cholesterol; TG: triglycerides; GRS_TC: genetic risk score for total cholesterol; GRS_LDL-c: genetic risk score for low-density lipoprotein cholesterol; GRS_HDL-c: genetic risk score for high-density lipoprotein cholesterol; GRS_TG: genetic risk score for triglycerides; TFAT: total body fat; VFAT: visceral fat; DR: drinkers; NDR: nondrinkers.

Moreover, estimations regarding the individual contribution of each independent predictor to blood lipid levels using PC^2^ are presented (Table 2). Interestingly, GRS_TC and GRS_LDL-c were the greatest contributors to TC and LDL-c features, respectively, with about 18% for both lipids (PC^2^ = 0.18). Likewise, VFAT and the respective GRS (GRS_HDL-c and GRS_TG) had a higher impact on HDL-c, with 9% (PC^2^ = 0.09) and 6% (PC^2^ = 0.06), respectively, as well as on TG concentrations, with 20% (PC^2^ = 0.20) and 7% (PC^2^ = 0.07), respectively. Additionally, comparisons of average blood lipid levels by predictor clusters based on median values are plotted (Figure 2). Greater differences in TC and LDL-c values were found by GRS_TC and GRS_LDL-c categorized by the median number of risk genotypes. Meanwhile, VFAT and the corresponding GRS categories (GRS_HDL-c and GRS_TG) accounted for higher variances in HDL-c and TG, respectively, as compared to other factors including energy intake, alcohol consumption, cholesterol intake, and TFAT (Figure 2).

## 4. Discussion

In the last years, multiple genetic variants have been found to be associated with specific phenotypes and metabolic disorders, including dyslipidemia [6]. In the current investigation, 45 obesity-related SNPs were associated with circulating lipids (TC, LDL-c, HDL-c, and TG). Of note, some of such associations are reported for the first time, except for rs1799883 (*FABP2*) [34], rs660339 (*UCP2*) and rs659366 (*UCP2*) [35], rs1052700 (*PLIN1*) [36], rs17782313 (*MC4R*) [37], rs7799039 (*LEP*) [38], rs2943641 (*IRS1*) [39], rs9939609 (*FTO*) [40], and rs324420 (*FAAH*) [41]. Interestingly, about 50% of SNPs were related to one specific circulating lipid, whereas only rs1685325 (*UCP3*) and rs894160 (*PLIN1*) were common among all lipids. This finding is consistent with previous studies illustrating the number of loci influencing blood lipid phenotypes using genome-wide and customized genotyping approaches [6, 42]. In contrast to UCP1, it has been postulated that UCP3 regulates cellular lipid metabolism by exporting those fatty acids that cannot be oxidized from the mitochondrial matrix to prevent their deleterious accumulation [43]. Meanwhile, PLIN1 is an adipocyte-specific lipid-coated protein involved in the regulation of lipolysis by regulating lipase interactions [44]. Also, PLIN1 promotes the efficient lipid droplet formation in adipocytes [45].

The magnitude of associations between individual gene variants and metabolic traits is generally modest. Therefore, effect size estimations based on the combination of multiple loci into a GRS are a common method to improve the predictive value of simple SNPs [46, 47]. In this study, GRS adding risk genotypes were major predictors of their respective plasma lipid in all performed linear regression models, mainly for TC and LDL-c blood concentrations (both 18%) and followed by TG (7%) and HDL-c (6%). Lower effects were reported for different GRS constructed from published meta-analyses of individuals of European ancestry, explaining 7%, 6%, 4%, and 3% of the total variance in HDL-c, TC, LDL-c, and TG, respectively [48]. Also, the combination of GWAS-identified or well-established lipid-related genetic loci into a weighted GRS explained no more than 11% of the blood lipid oscillations in major ethnic groups living in the United States, with no evidence of interactions between GRS and ethnicity [49]. In a cross-sectional study, 4 weighted GRS of lipid-associated SNPs accounted for 8% (TC), 7% (HDL-c), 6% (LDL-c), and 5% (TG) of the total variance in two Danish cohorts [50]. Furthermore, the highest quartile (more than 8 risk alleles) of a calculated GRS from obesity-predisposing variants was significantly associated with lower HDL-c levels compared to the lowest GRS quartile (lower than 4 risk alleles) in women with type 2 diabetes mellitus [51].

To date, most available studies analyzing the association of GRS with dyslipidemia and cardiovascular risk use an additive model of allele risk codification (0, 1, 2) across a number of genetic variants [48–51]. In this investigation, no additive effects in any included SNP were detected, so GRS were constructed according to different genotype categories. Interestingly, heterozygous genotypes of some SNPs were associated with the most favorable blood lipid phenotype compared to both homozygous groups, including rs8192678 (*PPARGC1A*), rs1052700 (*PLIN1*), rs894160 (*PLIN1*), rs7799039 (*LEP*), rs6567160 (*MC4R*), rs3813929 (*HTR2C*), rs11091046 (*AGTR2*), rs1386835 (*PPARG*), and rs1805081 (*NPC1*). This finding, known as heterozygote advantage, is a genetic condition in which heterozygous individuals for a locus have greater biological efficacy than the homozygous ones for the same locus [52]. Indeed, quantitative genetics theory predicts that this phenomenon, related to individual genetic diversity, should influence the variation in genetic predisposition to metabolic risks that show dominance variance. Therefore, it has been suggested that heterozygosity must be considered in genetic epidemiological studies concerning common disease traits [53].

Excessive adiposity is generally accompanied by unfavorable blood lipid patterns, which may depend upon regional fat distribution [54]. Here, VFAT has been associated with high TG levels but negatively correlated with HDL-c levels. Instead, TFAT increases tended to diminish circulating TG. In agreement with our findings, visceral adiposity has been shown to have a detrimental effect on plasma lipids, even after adjusting for abdominal subcutaneous adipose tissue [55]. For example, central fat accumulation showed a stronger association with metabolic risks than total fat mass in normal-weight Chinese adults [56].

Besides the genetic background, modifiable environmental factors may also influence serum lipids and related cardiovascular risk [5]. In this research, protein intake and alcohol drinking were positively associated with circulating HDL-c but negatively correlated with dietary cholesterol. Consistently, higher HDL-c concentrations have been reported in people consuming high-protein diets, accounting for a lower risk of developing cardiometabolic disease [57, 58]. Also, most available randomized-controlled trials have reported modest but significant increases in serum HDL-c concentrations after cholesterol supplementation with eggs [59]. Additionally, favorable lipid outcomes (higher levels of HDL-c) have been linked to moderate ethanol consumption, providing indirect evidence for a protective effect of alcohol on cardiovascular risk [60, 61].

The main strengths of this investigation include the analysis of the genetic influence on blood lipids using GRS from obesity-related SNPs instead of conventional lipid-protein genes as well as the use of different multiple linear regression tests to evaluate the contribution of genetic and lifestyle factors to plasma lipid profiles. Although SNPs were located on obesity-related genes, some of the genes also have a direct role in lipid metabolism including *PPARG*, *FABP2*, *PLIN1*, *NPC1*, *ACSL5*, and *FAAH*, suggesting relationships between genetics, adiposity, and plasma lipid profiles. Also, the results found in this research are unlikely to be confounded by population stratification since the studied sample was ethnically homogeneous (Spanish individuals of European ancestry) as revealed by AMOVA analyses. As for drawbacks, our findings may be not generalizable to other ethnic groups and populations, especially those who are exposed to different gene-environmental interactions. Moreover, this study enrolled subjects with excessive body weight-for-height; thus, further research is needed concerning the analysis of lean individuals. In addition, type I and type II errors cannot be completely ruled out, especially those related to the selection of SNPs to be introduced into the GRS. However, as previously reviewed [62], genomic profile risk scoring analyses can tolerate, at balance, some of these biases due to the use of less stringent *P* value thresholds compared to association studies of single variants. Likewise, although all linear regression models were internally validated by the bootstrapping method, it is not likely that the overfitting problem is totally ruled out. Also, because our findings were not assessed in an independent validation data set, replications in external populations may be required in a further study. Another way of validation could consist in splitting the original data set into two subsets, separating a discovery sample (training) and a target sample (testing), but given the relatively low sample analyzed, the statistical power of the study concerning main outcomes would be lowered. Furthermore, the role of new SNPs associated with excessive adiposity and accompanying metabolic alterations through a GRS approach needs to be explored. As a final point, while several gene-gene or gene-environment interactions in relation to lipid traits have been reported [5], no relevant relationships were found in this study.

In conclusion, our results suggest that multiple obesity-related genetic variants are important predictors of blood lipid phenotypes, in addition to environmental influences in subjects with excessive body weight-for-height. Together, these insights may contribute to design and implement precision lifestyle strategies to the control of lipid disorders.

## Figures and Tables

**Table 1:** Anthropometric, biochemical, and nutritional characteristics of the study population (*n* = 304).

Variable	Average values
Age (years)	45.8 ± 10.5
Sex (F/M)	212/92
*Anthropometrics and clinical data*	
Weight (kg)	87.7 ± 13.0
BMI (kg/m^2^)	31.6 ± 3.5
WC (cm)	102 ± 11
TFAT (kg)	36.9 ± 7.6
VFAT (kg)	1.48 ± 0.90
SBP (mmHg)	128 ± 17
DBP (mmHg)	79 ± 11
*Biochemical profile*	
Glucose (mg/dl)	96.6 ± 14.1
TC (mg/dl)	216 ± 38
HDL-c (mg/dl)	55.3 ± 12.9
LDL-c (mg/dl)	140 ± 34
TG (mg/dl)	104 ± 56
*Dietary intake/day*	
Energy (Kcal)	2970 ± 934
Carbohydrates (% E)	40.7 ± 6.8
Protein (% E)	17.0 ± 2.9
Fat (% E)	40.4 ± 5.8
*Lifestyle*	
Smokers (%)	21.9
Drinkers (%)	13.5
METs	23.8 ± 20.0

Variables are expressed as means ± standard deviations. BMI: body mass index; WC: waist circumference; TFAT: total body fat; VFAT: visceral fat; SBP: systolic blood pressure; DBP: diastolic blood pressure; TC: total cholesterol; HDL-c: high-density lipoprotein cholesterol; LDL-c: low-density lipoprotein cholesterol; TG: triglycerides; METs: metabolic equivalents.

**Table 2:** Best multiple linear regression models explaining blood lipid levels as dependent variables.

	TC		LDL-c		HDL-c		TG	
Predictors	*β*	PC^2^	*β*	PC^2^	*β*	PC^2^	*β*	PC^2^
Age (years)	0.80 ± 0.19	0.06	0.58 ± 0.18	0.04	0.22 ± 0.07	0.04		
Sex					4.51 ± 1.98	0.02		
Energy intake (100 kcal)	0.38 ± 0.21	0.01			0.18 ± 0.12	0.009		
Protein intake (%)					0.93 ± 0.30	0.04		
Cholesterol intake (mg)					−0.01 ± 0.004	0.02		
Alcohol					5.83 ± 2.05	0.03	−19.21 ± 9.33	0.02
TFAT (kg)							−1.14 ± 0.42	0.03
VFAT (kg)					−5.22 ± 1.05	0.09	30.95 ± 3.92	0.20
GRS_TC	6.55 ± 0.83	0.18						
GRS_LDL-c			6.79 ± 0.87	0.18				
GRS_HDL-c					−1.12 ± 0.27	0.06		
GRS_TG							4.20 ± 0.97	0.07
Constant	93.40 ± 12.99		45.74 ± 11.43		42.72 ± 7.71		61.58 ± 17.31	

*R* ^2^	0.2578		0.2217		0.3394		0.2828	
Adj. *R* ^2^	0.2501		0.2160		0.3192		0.2715	
Optimism correction coefficient for *R* ^2^	0.0112		0.0083		0.0373		0.0211	
Optimism correction coefficient for adj. *R* ^2^	0.0113		0.0084		0.0384		0.0214	
Optimism-corrected *R* ^2^	0.2466		0.2134		0.3021		0.2617	
Optimism-corrected adj. *R* ^2^	0.2388		0.2076		0.2808		0.2501	

Data are expressed as *β* values ± standard errors. The best models for each lipid phenotype were TC (BSRP, AIC/AICC); LDL-c (BSRP, BIC); HDL (BSRP, AICC); TG (BSM). BSRP: best subset regression procedure; AIC: akaike information criterion; AICC: corrected akaike information criterion; BIC: bayesian information criterion; BSM: bootstrapping stepwise method; PC^2^: squared partial correlation; TC: total cholesterol; LDL-c: low-density lipoprotein cholesterol; HDL-c: high-density lipoprotein cholesterol; TG: triglycerides; TFAT: total body fat; VFAT: visceral fat; GRS_TC: genetic risk score for total cholesterol; GRS_LDL-c: genetic risk score for low-density lipoprotein cholesterol; GRS_HDL-c: genetic risk score for high-density lipoprotein cholesterol; GRS_TG: genetic risk score for triglycerides.

## Data Availability

The data used to support the findings of this study are included within the article.

## Supplementary Materials

Supplementary Materials Supplementary Table 1: genomic characteristics of the 95 obesity-predisposing SNPs. Supplementary Table 2a: list of SNPs associated with circulating total cholesterol levels and related genotype codifications. Supplementary Table 2b: list of SNPs associated with circulating LDL-c levels and related genotype codifications. Supplementary Table 2c: list of SNPs associated with circulating HDL-c levels and related genotype codifications. Supplementary Table 2d: list of SNPs associated with circulating triglyceride levels and related genotype codifications. Supplementary Table 3a: multiple linear regression models explaining total cholesterol levels as the dependent variable. Supplementary Table 3b: multiple linear regression models explaining LDL-c levels as the dependent variable. Supplementary Table 3c: multiple linear regression models explaining HDL-c levels as the dependent variable. Supplementary Table 3d: multiple linear regression models explaining triglyceride levels as the dependent variable.
