# Supplementary material for: Prediction of Blood Lipid Phenotypes Using Obesity-Related Genetic Polymorphisms and Lifestyle Data in Subjects with Excessive Body Weight
Source: Int J Genomics. 2018 Nov 19;2018:4283078. doi: 10.1155/2018/4283078 (PMC6276413; doi:10.1155/2018/4283078)
Supplement: Supplementary Materials — Supplementary Table 1: genomic characteristics of the 95 obesity-predisposing SNPs. Supplementary Table 2a: list of SNPs associated with circulating total cholesterol levels and related genotype codifications. Supplementary Table 2b: list of SNPs associated with circulating LDL-c levels and related genotype codifications. Supplementary Table 2c: list of SNPs associated with circulating HDL-c levels and related genotype codifications. Supplementary Table 2d: list of SNPs associated with circulating triglyceride levels and related genotype codifications. Supplementary Table 3a: multiple linear regression models explaining total cholesterol levels as the dependent variable. Supplementary Table 3b: multiple linear regression models explaining LDL-c levels as the dependent variable. Supplementary Table 3c: multiple linear regression models explaining HDL-c levels as the dependent variable. Supplementary Table 3d: multiple linear regression models explaining triglyceride levels as the dependent variable. [file 4283078.f1.docx]

**Supplementary Table 1.** Genomic characteristics of the 95 obesity-predisposing SNPs.

| Gene | SNP | Chromosome position | Alleles | MAF^1^ | MAF^2^ | HWE |
| --- | --- | --- | --- | --- | --- | --- |
| *GNAT2* | rs17024393 | Chr1:110154688 | T/C | 0.019 (C) | 0.016 (C) | **0.087** |
| *MTHFR* | rs1801131 | Chr1:11854476 | T/G | 0.271 (G) | 0.260 (G) | 1.000 |
| *MTHFR* | rs1801133 | Chr1:11856378 | G/A | 0.444 (A) | 0.387 (A) | 0.375 |
| *SEC16B* | rs543874 | Chr1:177889480 | A/G | 0.145 (G) | 0.139 (G) | 1.000 |
| *LYPLAL1* | rs2605100 | Chr1:219644224 | A/G | 0.318 (A) | 0.281 (A) | 0.724 |
| *LYPLAL1* | rs4846567 | Chr1:219750717 | G/T | 0.313 (T) | 0.303 (T) | 0.733 |
| *CNR2* | rs3123554 | Chr1:24196401 | A/G | 0.439 (A) | 0.511 (A) | 1.000 |
| *FAAH* | rs324420 | Chr1:46870761 | C/A | 0.164 (A) | 0.196 (A) | 0.825 |
| *LEPR* | rs8179183/rs1805094 | Chr1:66075952 | G/C | 0.112 (C) | 0.155 (C) | 0.583 |
| *NEGR1* | rs2815752 | Chr1:72812440 | G/A | 0.341 (G) | 0.344 (G) | 0.217 |
| *ABCB11* | rs519887 | Chr2:169780885 | T/C | 0.542 (C) | 0.447 (C) | 0.254 |
| *ABCB11* | rs484066 | Chr2:169782481 | A/T | 0.355 (A) | 0.406 (A) | 0.369 |
| *ABCB11* | rs569805 | Chr2:169782880 | A/T | 0.355 (A) | 0.370 (A) | 0.435 |
| *ABCB11* | rs494874 | Chr2:169789306 | T/C | 0.346 (T) | 0.362 (T) | 0.532 |
| *IRS1* | rs2943641 | Chr2:227093745 | T/C | 0.388 (T) | 0.354 (T) | 1.000 |
| *ADCY3* | rs10182181 | Chr2:25150296 | A/G | 0.491 (A) | 0.503 (A) | 0.160 |
| *ADCY3* | rs713586 | Chr2:25158008 | T/C | 0.481 (T) | 0.502 (T) | 0.206 |
| *TMEM18* | rs2860323 | Chr2:614210 | A/G | 0.140 (A) | 0.183 (A) | 0.813 |
| *TMEM18* | rs2867125 | Chr2:622827 | T/C | 0.145 (T) | 0.208 (T) | 1.000 |
| *TMEM18* | rs13021737 | Chr2:632348 | A/G | 0.150 (A) | 0.180 (A) | 1.000 |
| *PPARG* | rs1801282 | Chr3:12393125 | C/G | 0.117 (G) | 0.081 (G) | 1.000 |
| *PPARG* | rs2959272 | Chr3:12442833 | T/G | 0.523 (T) | 0.507 (T) | 0.482 |
| *PPARG* | rs1386835 | Chr3:12450918 | A/G | NA | 0.243 (G) | **<0.001** |
| *PPARG* | rs709158 | Chr3:12463176 | A/G | 0.341 (G) | 0.318 (G) | 0.873 |
| *PPARG* | rs1175540 | Chr3:12465243 | C/A | 0.346 (A) | 0.320 (A) | 1.000 |
| *PPARG* | rs1175544 | Chr3:12467044 | C/T | 0.327 (T) | 0.321 (T) | 0.526 |
| *PPARG* | rs1797912 | Chr3:12470239 | A/C | 0.374 (C) | 0.350 (C) | 0.878 |
| *ETV5* | rs1516725 | Chr3:185824004 | T/C | 0.173 (T) | 0.109 (T) | 0.436 |
| *ETV5* | rs9816226 | Chr3:185834499 | A/T | 0.234 (A) | 0.174 (A) | 0.439 |
| *SLC39A8* | rs13107325 | Chr4:103188709 | C/A/T | 0.084 (T) | 0.070 (T) | 0.606 |
| *FABP2* | rs1799883 | Chr4:120241902 | T/C | 0.271 (T) | 0.296 (T) | 0.385 |
| *UCP1* | rs6536991 | Chr4:141481581 | T/C | 0.322 (C) | 0.230 (C) | **0.099** |
| *UCP1* | rs12502572 | Chr4:141485134 | G/A | 0.621 (G) | 0.692 (G) | 0.492 |
| *UCP1* | rs1800592 | Chr4:141493961 | T/C | 0.257 (C) | 0.192 (C) | 0.338 |
| *PPARGC1A* | rs8192678 | Chr4:23815662 | C/T | 0.388 (T) | 0.337 (T) | 0.199 |
| *GNPDA2* | rs10938397 | Chr4:45182527 | A/G | 0.374 (G) | 0.473 (G) | **0.051** |
| *CLOCK* | rs1801260 | Chr4:56301369 | A/G | 0.294 (G) | 0.268 (G) | 0.369 |
| *PPM1K* | rs1440581 | Chr4:89226422 | T/C | 0.430 (T) | 0.454 (T) | 0.776 |
| *ADRB2* | rs1042713 | Chr5:148206440 | G/A | 0.383 (A) | 0.370 (A) | **0.068** |
| *ADRB2* | rs1042714 | Chr5:148206473 | G/C | 0.425 (G) | 0.566 (G) | **<0.001** |
| *CPEB4* | rs6861681 | Chr5:173362458 | G/A | 0.206 (A) | 0.271 (A) | 0.391 |
| *TNFA* | rs1800629 | Chr6:31543031 | G/A | 0.145 (A) | 0.118 (A) | 1.000 |
| *NUDT3* | rs206936 | Chr6:34302869 | A/G | 0.210 (G) | 0.235 (G) | 0.444 |
| *TFAP2B* | rs987237 | Chr6:50803050 | A/G | 0.145 (G) | 0.166 (G) | 0.609 |
| *TFAP2B* | rs2207139 | Chr6:50845490 | A/G | 0.131 (G) | 0.155 (G) | 0.427 |
| *LEP* | rs7799039 | Chr7:127878783 | G/A | 0.388 (A) | 0.516 (A) | 0.887 |
| *LEP* | rs4731426 | Chr7:127882070 | G/C | 0.561 (C) | 0.623 (C) | 0.543 |
| *LEP* | rs2071045 | Chr7:127892980 | T/C | 0.313 (C) | 0.250 (C) | 0.849 |
| *NFE2L3* | rs1055144 | Chr7:25871109 | C/T | 0.192 (T) | 0.168 (T) | 0.306 |
| *ADRB3* | rs4994 | Chr8:37823798 | A/G | 0.061 (G) | 0.082 (G) | 0.371 |
| *ADRA2A* | rs1800544 | Chr10:112836503 | G/C | 0.729 (C) | 0.706 (C) | 0.611 |
| *ACSL5* | rs2419621 | Chr10:114135013 | C/T | 0.276 (T) | 0.278 (T) | 0.149 |
| *TCF7L2* | rs7903146 | Chr10:114758349 | C/T | 0.397 (T) | 0.360 (T) | 1.000 |
| *TCF7L2* | rs12255372 | Chr10:114808902 | G/T | 0.374 (T) | 0.368 (T) | 0.651 |
| *ANKK1* | rs1800497 | Chr11:113270828 | G/A | 0.145 (A) | 0.191 (A) | 1.000 |
| *APOA5* | rs662799 | Chr11:116663707 | G/A | 0.093 (G) | 0.071 (G) | 1.000 |
| *BDNF* | rs6265 | Chr11:27679916 | C/T | 0.210 (T) | 0.212 (T) | 1.000 |
| *BDNF* | rs11030104 | Chr11:27684517 | A/G | 0.238 (G) | 0.234 (G) | 0.556 |
| *BDNF* | rs10767664 | Chr11:27725986 | T/A | 0.266 (T) | 0.264 (T) | 0.212 |
| *CRY2* | rs11605924 | Chr11:45873091 | A/C | 0.472 (C) | 0.470 (C) | 0.888 |
| *MTCH2* | rs10838738 | Chr11:47663049 | A/G | 0.360 (G) | 0.373 (G) | **0.074** |
| *UCP2* | rs660339 | Chr11:73689104 | G/A | 0.364 (A) | 0.393 (A) | 0.883 |
| *UCP2* | rs659366 | Chr11:73694754 | C/T | 0.332 (T) | 0.363 (T) | 0.756 |
| *UCP3* | rs2075577 | Chr11:73715542 | G/A | 0.579 (G) | 0.451 (G) | 0.887 |
| *UCP3* | rs2734827 | Chr11:73716277 | G/A | 0.308 (A) | 0.349 (A) | 1.000 |
| *UCP3* | rs1685325 | Chr11:73717025 | T/C | 0.598 (T) | 0.508 (T) | 0.889 |
| *UCP3* | rs2075576 | Chr11:73717121 | C/T | 0.164 (T) | 0.176 (T) | 0.649 |
| *UCP3* | rs1800006 | Chr11:73717254 | A/G | 0.173 (G) | 0.184 (G) | 0.368 |
| *UCP3* | rs1800849 | Chr11:73720165 | G/A | 0.164 (A) | 0.174 (A) | 0.642 |
| *STK33* | rs4929949 | Chr11:8604593 | T/C | 0.481 (C) | 0.536 (C) | 0.325 |
| *MTNR1B* | rs10830963 | Chr11:92708710 | C/G | 0.276 (G) | 0.273 (G) | 0.180 |
| *ALOX5AP* | rs4769873 | Chr13:31312689 | C/T | 0.079 (T) | 0.109 (T) | 0.713 |
| *PLIN1* | rs1052700 | Chr15:90208310 | A/T | 0.308 (T) | 0.326 (T) | 0.870 |
| *PLIN1* | rs894160 | Chr15:90211823 | C/T | 0.280 (T) | 0.275 (T) | 1.000 |
| *PLIN1* | rs2289487 | Chr15:90217096 | C/T | 0.654 (T) | 0.668 (T) | 0.749 |
| *SH2B1* | rs7498665 | Chr16:28883241 | A/G | 0.294 (G) | 0.311 (G) | **0.064** |
| *SH2B1* | rs7359397 | Chr16:28885659 | C/T | 0.290 (T) | 0.306 (T) | **0.062** |
| *FTO* | rs1558902 | Chr16:53803574 | T/A | 0.393 (A) | 0.413 (A) | 0.186 |
| *FTO* | rs1121980 | Chr16:53809247 | G/A | 0.411 (A) | 0.416 (A) | 0.375 |
| *FTO* | rs17817449 | Chr16:53813367 | T/G | 0.374 (G) | 0.387 (G) | 0.762 |
| *FTO* | rs8050136 | Chr16:53816275 | C/A | 0.374 (A) | 0.387 (A) | 0.761 |
| *FTO* | rs3751812 | chr16:53818460 | G/T | 0.374 (T) | 0.380 (T) | 0.651 |
| *FTO* | rs9939609 | Chr16:53820527 | T/A | 0.374 (A) | 0.388 (A) | 0.881 |
| *AANAT* | rs12452844 | Chr17:74459243 | G/A | 0.276 (A) | 0.258 (A) | 0.192 |
| *NPC1* | rs1805081 | Chr18:21140432 | T/C | 0.355 (C) | 0.332 (C) | 0.273 |
| *MC4R* | rs6567160 | Chr18:57829135 | T/C | 0.257 (C) | 0.219 (C) | 0.136 |
| *MC4R* | rs571312 | Chr18:57839769 | C/A | 0.257 (A) | 0.217 (A) | 0.127 |
| *MC4R* | rs17782313 | Chr18:57851097 | T/C | 0.257 (C) | 0.045 (C) | **<0.001** |
| *MC4R* | rs17066866 | Chr18:58055619 | A/T | 0.009 (T) | 0.019 (T) | 1.000 |
| *TNFRSF11A* | rs17069904 | Chr18:60032949 | G/A | 0.126 (A) | 0.107 (A) | 0.701 |
| *QPCTL* | rs2287019 | Chr19:46202172 | C/T | 0.173 (T) | 0.143 (T) | **0.016** |
| *CTNNBL1* | rs6013029 | Chr20:36399580 | G/T | 0.107 (T) | 0.090 (T) | 0.648 |
| *GNAS* | rs6123837 | Chr20:57465571 | G/A | 0.388 (A) | 0.411 (A) | 0.883 |
| *HTR2C* | rs3813929 | ChrX:113818520 | C/T | 0.156 (T) | 0.179 (T) | **<0.001** |
| *AGTR2* | rs11091046 | ChrX:115305126 | A/C | 0.412 (A) | 0.441 (A) | 0.104 |

MAF^1^: Minor Allele Frequency from 1000 Genomes database concerning Iberian (Spanish) population; MAF^2^: Minor Allele Frequency regarding this study population; HWE: Hardy-Weinberg equilibrium; NA: not available. MAF are reported concerning Iberian (Spanish) population. HWE is expressed as *P* value.

**Supplementary Table 2a.** List of SNPs associated with circulating total cholesterol levels and related genotype codifications.

|  | Alleles | Genotype codification | | n | | Means ± SD | |  |
| --- | --- | --- | --- | --- | --- | --- | --- | --- |
| SNP (gene) |  | 0 | 1 | 0 | 1 | 0 | 1 | *P* value |
| rs2605100 (*LYPLAL1*) | A/G | AG+GG | AA | 284 | 18 | 214.8±38.1 | 233.6±37.5 | **0.043** |
| rs569805 (*ABCB11*) | A/T | AA | AT+TT | 42 | 260 | 205.2±34.1 | 217.6±38.7 | 0.050 |
| rs494874 (*ABCB11*) | T/C | TT | TC+CC | 39 | 263 | 205.2±34.4 | 217.5±38.6 | 0.061 |
| rs1175544 (*PPARG*) | C/T | CT+TT | CC | 160 | 142 | 212.2±40.7 | 220.1±35.1 | 0.074 |
| rs1797912 (*PPARG*) | A/C | AC+CC | AA | 173 | 128 | 212.0±40.2 | 220.8±34.9 | **0.047** |
| rs1799883 (*FABP2*) | T/C | TC+CC | TT | 274 | 28 | 214.5±38.6 | 229.7±32.8 | **0.045** |
| rs6536991 (*UCP1*) | T/C | CC | TT+TC | 22 | 280 | 195.0±27.5 | 217.5±38.5 | **0.008** |
| rs12502572 (*UCP1*) | G/A | AA | GG+GA | 33 | 269 | 194.8±27.3 | 218.5±38.7 | **0.001** |
| rs8192678 (*PPARGC1A*) | C/T | CT | CC+TT | 123 | 179 | 210.4±38.3 | 219.7±37.9 | **0.040** |
| rs1801260 (*CLOCK*) | A/G | AG+GG | AA | 139 | 163 | 210.7±35.6 | 220.3±40.0 | **0.030** |
| rs1440581 (*PPM1K*) | T/C | TT+TC | CC | 214 | 88 | 213.2±39.1 | 222.5±35.5 | 0.054 |
| rs206936 (*NUDT3*) | A/G | GG | AA+AG | 16 | 286 | 198.3±32.7 | 216.9±38.4 | 0.058 |
| rs10838738 (*MTCH2*) | A/G | GG | AA+AG | 50 | 252 | 201.3±34.7 | 218.8±38.3 | **0.003** |
| rs660339 (*UCP2*) | G/A | GG+GA | AA | 256 | 46 | 213.7±37.9 | 228.1±38.2 | **0.018** |
| rs659366 (*UCP2*) | C/T | CC+CT | TT | 261 | 41 | 214.2±38.0 | 227.0±38.4 | **0.045** |
| rs1685325 (*UCP3*) | T/C | TT+CC | TC | 148 | 154 | 211.1±36.4 | 220.5±39.5 | **0.033** |
| rs4929949 (*STK33*) | T/C | TT+CC | TC | 155 | 147 | 209.6±35.6 | 222.6±40.0 | **0.003** |
| rs1052700 (*PLIN1*) | A/T | AT | AA+TT | 131 | 171 | 209.8±38.1 | 220.6±37.8 | **0.015** |
| rs894160 (*PLIN1*) | C/T | CT | CC+TT | 129 | 173 | 211.5±37.5 | 219.2±38.6 | 0.087 |
| rs12452844 (*AANAT*) | G/A | AA | GG+GA | 16 | 281 | 199.1±32.2 | 216.9±38.4 | 0.070 |
| rs17782313 (*MC4R*) | T/C | TT | TC+CC | 283 | 17 | 214.9±37.5 | 232.4±46.9 | 0.068 |
| rs6013029 (*CTNNBL1*) | G/T | GG+TT | GT | 254 | 48 | 214.1±38.0 | 225.3±38.6 | 0.062 |

Variables are expressed as means ± standard deviations. 0 = non-risk genotype; 1 = risk genotype. Values in bold (p<0.05).

**Supplementary Table 2b.** List of SNPs associated with circulating LDL-c levels and related genotype codifications.

|  | Alleles | Genotype codification | | n | | Means ± SD | |  |
| --- | --- | --- | --- | --- | --- | --- | --- | --- |
| SNP (gene) |  | 0 | 1 | 0 | 1 | 0 | 1 | *P* value |
| rs2605100 (*LYPLAL1*) | A/G | AG+GG | AA | 284 | 18 | 138.8±33.4 | 155.8±35.8 | **0.039** |
| rs1799883 (*FABP2*) | T/C | TC+CC | TT | 274 | 28 | 138.7±33.8 | 151.0±31.3 | 0.066 |
| rs6536991 (*UCP1*) | T/C | CC | TT+TC | 22 | 280 | 123.7±25.8 | 141.1±34.0 | **0.019** |
| rs12502572 (*UCP1*) | G/A | AA | GG+GA | 33 | 269 | 121.4±25.1 | 142.1±34.0 | **0.001** |
| rs1440581 (*PPM1K*) | T/C | TT+TC | CC | 214 | 88 | 137.7±34.8 | 145.1±30.6 | 0.082 |
| rs206936 (*NUDT3*) | A/G | GG | AA+AG | 16 | 286 | 125.7±27.9 | 140.6±33.9 | 0.086 |
| rs7799039 (*LEP*) | G/A | GA | GG+AA | 146 | 153 | 135.8±32.9 | 143.4±34.5 | 0.053 |
| rs10838738 (*MTCH2*) | A/G | GG | AA+AG | 50 | 252 | 126.1±30.5 | 142.6±33.8 | **0.001** |
| rs1685325 (*UCP3*) | T/C | TT+CC | TC | 148 | 154 | 135.7±32.3 | 143.8±34.7 | **0.038** |
| rs4929949 (*STK33*) | T/C | TT+CC | TC | 155 | 147 | 134.5±31.7 | 145.4±35.0 | **0.005** |
| rs1052700 (*PLIN1*) | A/T | AT+TT | AA | 164 | 138 | 135.6±33.5 | 144.8±33.5 | **0.018** |
| rs894160 (*PLIN1*) | C/T | CT | CC+TT | 129 | 173 | 135.4±32.9 | 143.1±34.1 | **0.049** |
| rs7498665 (*SH2B1*) | A/G | AA | AG+GG | 148 | 153 | 136.3±33.0 | 143.4±34.3 | 0.069 |
| rs7359397 (*SH2B1*) | C/T | CC | CT+TT | 150 | 151 | 136.1±32.8 | 143.8±34.3 | **0.049** |
| rs12452844 (*AANAT*) | G/A | AA | GG+GA | 16 | 281 | 124.0±27.7 | 140.8±33.8 | 0.052 |
| rs1805081 (*NPC1*) | T/C | TT+CC | TC | 181 | 121 | 137.0±33.2 | 144.0±34.3 | 0.078 |
| rs17782313 (*MC4R*) | T/C | TT | TC+CC | 283 | 17 | 138.9±33.3 | 156.3±38.3 | **0.038** |
| rs3813929 (*HTR2C*) | C/T | CT+TT | CC | 84 | 218 | 133.4±34.6 | 142.3±33.2 | **0.041** |
| rs11091046 (*AGTR2*) | A/C | AC+CC | AA | 216 | 71 | 138.1±33.6 | 146.9±34.1 | 0.057 |

Variables are expressed as means ± standard deviations. 0 = non-risk genotype; 1 = risk genotype. Values in bold (p<0.05).

**Supplementary Table 2c.** List of SNPs associated with circulating HDL-c levels and related genotype codifications.

|  | Alleles | Genotype codification | | n | | Means ± SD | |  |
| --- | --- | --- | --- | --- | --- | --- | --- | --- |
| SNP (gene) |  | 0 | 1 | 0 | 1 | 0 | 1 | *P* value |
| rs2815752 (*NEGR1*) | G/A | GG+AA | GA | 156 | 146 | 56.7±14.2 | 53.9±11.4 | 0.058 |
| rs2943641 (*IRS1*) | T/C | TT | TC+CC | 40 | 262 | 58.8±13.0 | 54.8±12.9 | 0.066 |
| rs8192678 (*PPARGC1A*) | C/T | CC+TT | CT | 179 | 123 | 56.5±13.2 | 53.7±12.4 | 0.069 |
| rs2419621 (*ACSL5*) | C/T | CC+CT | TT | 280 | 22 | 55.8±13.0 | 49.6±10.3 | **0.031** |
| rs6265 (*BDNF*) | C/T | CC+CT | TT | 291 | 11 | 55.7±12.9 | 45.3±9.0 | **0.009** |
| rs11030104 (*BDNF*) | A/G | AA+AG | GG | 286 | 16 | 55.7±13.0 | 48.0±9.7 | **0.019** |
| rs660339 (*UCP2*) | G/A | GG+AA | GA | 156 | 146 | 56.6±14.1 | 54.0±11.4 | 0.073 |
| rs659366 (*UCP2*) | C/T | CC+TT | CT | 164 | 138 | 56.7±14.0 | 53.7±11.5 | **0.046** |
| rs2075577 (*UCP3*) | G/A | GG | GA+AA | 63 | 239 | 58.5±13.7 | 54.5±12.6 | **0.027** |
| rs1685325 (*UCP3*) | T/C | TT | TC+CC | 77 | 225 | 59.3±13.1 | 54.0±12.6 | **0.002** |
| rs4769873 (*ALOX5AP*) | C/T | CC | CT+TT | 240 | 62 | 56.5±12.9 | 51.0±12.2 | **0.003** |
| rs894160 (*PLIN1*) | C/T | CC+TT | CT | 173 | 129 | 56.6±12.5 | 53.6±13.4 | **0.047** |
| rs2289487 (*PLIN*1) | C/T | CC+TT | CT | 162 | 139 | 56.8±12.8 | 53.7±12.9 | **0.040** |
| rs9939609 (*FTO*) | T/A | TT+TA | AA | 255 | 47 | 55.9±13.0 | 52.1±12.3 | 0.061 |
| rs6567160 (*MC4R*) | T/C | TC | TT+CC | 97 | 205 | 57.3±14.2 | 54.4±12.2 | 0.065 |
| rs2287019 (*QPCTL*) | C/T | CC | CT | 207 | 84 | 56.2±13.4 | 53.2±11.5 | 0.069 |
| rs3813929 (*HTR2C*) | C/T | CT | CC+TT | 61 | 241 | 57.9±11.3 | 54.7±13.3 | 0.079 |
| rs11091046 (*AGTR2*) | A/C | AC | AA+CC | 111 | 176 | 59.2±13.5 | 53.5±12.2 | **<0.001** |

Variables are expressed as means ± standard deviations. 0 = non-risk genotype; 1 = risk genotype. Values in bold (p<0.05).

**Supplementary Table 2d.** List of SNPs associated with circulating triglyceride levels and related genotype codifications.

|  | Alleles | Genotype codification | | n | | Means ± SD | |  |
| --- | --- | --- | --- | --- | --- | --- | --- | --- |
| SNP (gene) |  | 0 | 1 | 0 | 1 | 0 | 1 | *P* value |
| rs324420 (*FAAH*) | C/A | CC+AA | CA | 212 | 90 | 96.9±46.1 | 119.6±73.3 | **0.001** |
| rs2959272 (*PPARG*) | T/G | TT+TG | GG | 226 | 76 | 98.0±51.6 | 120.6±66.5 | **0.002** |
| rs1386835 (*PPARG*) | A/G | AG | AA | 148 | 134 | 97.1±51.2 | 113.1±63.6 | **0.021** |
| rs709158 (*PPARG*) | A/G | AG+GG | AA | 160 | 141 | 97.7±50.5 | 109.7±61.6 | 0.064 |
| rs1175540 (*PPARG*) | C/A | CA+AA | CC | 161 | 140 | 97.4±50.4 | 111.2±62.1 | **0.034** |
| rs1175544 (*PPARG*) | C/T | TT | CC+CT | 35 | 267 | 86.3±38.6 | 106.0±58.1 | 0.053 |
| rs1797912 (*PPARG*) | A/C | AC+CC | AA | 173 | 128 | 98.9±53.8 | 110.4±59.5 | 0.079 |
| rs1800544 (*ADRA2A*) | G/C | GG+GC | CC | 148 | 154 | 97.8±39.6 | 109.3±68.6 | 0.075 |
| rs660339 (*UCP2*) | G/A | GG | GA+AA | 110 | 192 | 94.9±39.2 | 108.7±63.8 | **0.040** |
| rs659366 (*UCP2*) | C/T | CC | CT+TT | 123 | 179 | 93.7±38.7 | 110.5±65.2 | **0.010** |
| rs2075577 (*UCP3*) | G/A | GG | GA+AA | 63 | 239 | 87.8±32.2 | 107.9±60.6 | **0.012** |
| rs1685325 (*UCP3*) | T/C | TT+CC | TC | 148 | 154 | 93.7±50.1 | 113.3±60.6 | **0.002** |
| rs894160 (*PLIN1*) | C/T | CC+TT | CT | 173 | 129 | 97.1±42.8 | 112.5±70.0 | **0.018** |
| rs2289487 (*PLIN1*) | C/T | TT | CC+CT | 132 | 169 | 94.8±42.7 | 109.6±63.6 | **0.022** |
| rs12452844 (*AANAT*) | G/A | AA | GG+GA | 16 | 281 | 72.0±19.6 | 105.9±57.6 | **0.020** |
| rs1805081 (*NPC1*) | T/C | TC | TT+CC | 121 | 181 | 96.4±49.0 | 108.5±60.6 | 0.067 |
| rs3813929 (*HTR2C*) | C/T | CC+CT | TT | 279 | 23 | 101.8±53.4 | 125.9±83.9 | **0.049** |
| rs11091046 (*AGTR2*) | A/C | AC | AA+CC | 111 | 176 | 92.7±44.3 | 110.7±63.4 | **0.009** |

Variables are expressed as means ± standard deviations. 0 = non-risk genotype; 1 = risk genotype. Values in bold (p<0.05).

**Supplementary Table 3a.** Multiple linear regression models explaining total cholesterol levels as dependent variable.

|  |  | BSRP | | |  |
| --- | --- | --- | --- | --- | --- |
| Predictors | LARS | Adj. R^2^ | **AIC/AICC** | BIC | BSM |
| Age (years) | 0.80±0.19 | 0.82±0.19 | **0.80±0.19** | 0.83±0.19 | 0.79±0.19 |
| Sex | -6.10±4.21 | -6.12±4.21 |  |  |  |
| Energy intake (100 kcal) | 0.33±0.21 | 0.35±0.21 | **0.38±0.21** |  | 0.40±0.21 |
| GRS_TC | 6.64±0.83 | 6.70±0.83 | **6.55±0.83** | 6.55±0.83 | 6.56±0.83 |
| BMI (kg/m^2)^ |  | -0.68±0.56 |  |  |  |
| Fat intake (%) |  |  |  |  | -0.24±0.33 |
| Constant | 98.20±13.38 | 117.67±20.78 | **93.40±12.99** | 103.57±11.77 | 102.99±18.77 |
| R^2^ | 0.2631 | 0.2669 | **0.2578** | 0.2493 | 0.2591 |
| Adj. R^2^ | 0.2529 | 0.2542 | **0.2501** | 0.2441 | 0.2488 |
| Optimism correction coefficient for R^2^ | 0.0163 | 0.0212 | **0.0112** | 0.0055 | 0.0158 |
| Optimism correction coefficient for adj. R^2^ | 0.0165 | 0.0216 | **0.0113** | 0.0055 | 0.0160 |
| Optimism-corrected R^2^ | 0.2468 | 0.2457 | **0.2466** | 0.2438 | 0.2433 |
| Optimism-corrected adj. R^2^ | 0.2364 | 0.2326 | **0.2388** | 0.2386 | 0.2328 |

Data are expressed as β values ± standard errors. TC: total cholesterol; BMI: body mass index; GRS_TC: genetic risk score for total cholesterol; LARS: least-angle regression; BSRP: best subset regression procedure; BSM: bootstrapping stepwise method; AIC: akaike information criterion; AICC: corrected akaike information criterion; BIC: bayesian information criterion. Values in bold indicate the best predictive model.

**Supplementary Table 3b.** Multiple linear regression models explaining LDL-c levels as dependent variable.

|  |  | BSRP | | |  |
| --- | --- | --- | --- | --- | --- |
| Predictors | LARS | Adj. R^2^ | AIC/AICC | **BIC** | BSM |
| Age (years) | 0.54±0.18 | 0.54±0.18 | 0.55±0.18 | **0.58±0.18** | 0.55±0.18 |
| Sex | -7.01±4.02 | -7.01±4.02 | 7.67±3.97 |  | 7.67±3.97 |
| Energy intake (100 kcal) | 0.20±0.19 | 0.20±0.19 |  |  |  |
| GRS_LDL-c | 6.60±0.88 | 6.60±0.88 | 6.70±0.87 | **6.79±0.87** | 6.70±0.87 |
| Fat intake (%) | -0.46±0.31 | -0.46±0.31 | 0.43±0.31 |  | 0.43±0.31 |
| Constant | 67.07±17.83 | 67.07±17.83 | 70.99±17.40 | **45.74±11.43** | 70.99±17.40 |
| R^2^ | 0.2413 | 0.2413 | 0.2384 | **0.2217** | 0.2384 |
| Adj. R^2^ | 0.2272 | 0.2272 | 0.2272 | **0.2160** | 0.2272 |
| Optimism correction coefficient for R^2^ | 0.0270 | 0.0270 | 0.0202 | **0.0083** | 0.0202 |
| Optimism correction coefficient for adj. R^2^ | 0.0275 | 0.0275 | 0.0205 | **0.0084** | 0.0205 |
| Optimism-corrected R^2^ | 0.2143 | 0.2143 | 0.2182 | **0.2134** | 0.2182 |
| Optimism-corrected adj. R^2^ | 0.1997 | 0.1997 | 0.2067 | **0.2076** | 0.2067 |

Data are expressed as β values ± standard errors. LDL-c: low-density lipoprotein cholesterol; GRS_LDL-c: genetic risk score for low-density lipoprotein cholesterol; LARS: least-angle regression; BSRP: best subset regression procedure; BSM: bootstrapping stepwise method; AIC: akaike information criterion; AICC: corrected akaike information criterion; BIC: bayesian information criterion. Values in bold indicate the best predictive model.

**Supplementary Table 3c.** Multiple linear regression models explaining HDL-c levels as dependent variable.

|  |  | BSRP | | | |  |
| --- | --- | --- | --- | --- | --- | --- |
| Predictors | LARS | Adj. R^2^ | AIC | **AICC** | BIC | BSM |
| Age (years) | 0.24±0.08 | 0.24±0.08 | 0.23±0.07 | **0.22±0.07** | 0.26±0.07 | 0.22±0.07 |
| Sex | 4.22±2.36 | 4.22±2.36 | 4.36±1.98 | **4.51±1.98** |  | 4.91±2.13 |
| Smoke | -1.96±1.63 | -1.96±1.63 |  |  |  |  |
| Alcohol | 3.60±2.39 | 3.60±2.39 | 5.32±2.08 | **5.83±2.05** |  | 5.15±2.10 |
| METs | 0.04±0.03 | 0.04±0.03 |  |  |  |  |
| Energy intake (100 kcal) | 0.22±0.12 | 0.22±0.12 | 0.21±0.12 | **0.18±0.12** |  | 0.21±0.12 |
| Carbohydrate intake (%) | -0.42±0.29 | -0.42±0.29 | -0.15±0.11 |  |  | -0.15±0.11 |
| Fat intake (%) | -0.33±0.31 | -0.33±0.31 |  |  |  |  |
| Protein intake (%) | 0.64±0.39 | 0.64±0.39 | 0.85±0.30 | **0.93±0.30** |  | 0.88±0.30 |
| Cholesterol intake (mg) | -0.01±0.004 | -0.01±0.004 | -0.01±0.004 | **-0.01±0.004** |  | -0.01±0.004 |
| BMI (kg/m^2^) | -0.68±0.41 | -0.68±0.41 |  |  |  | -0.17±0.24 |
| TFAT (kg) | 0.29±0.19 | 0.29±0.19 |  |  |  |  |
| VFAT (kg) | -4.91±1.34 | -4.91±1.34 | -5.38±1.06 | **-5.22±1.05** | -6.36±0.85 | -4.84±1.31 |
| GRS_HDL-c | -1.02±0.28 | -1.02±0.28 | -1.10±0.27 | **-1.12±0.27** | -1.27±0.27 | -1.11±0.27 |
| Constant | 86.17±30.21 | 86.17±30.21 | 50.11±9.34 | **42.72±7.71** | 63.67±3.71 | 53.96±10.82 |
| R^2^ | 0.3535 | 0.3535 | 0.3443 | **0.3394** | 0.2855 | 0.3456 |
| Adj. R^2^ | 0.3174 | 0.3174 | 0.3216 | **0.3192** | 0.2774 | 0.3203 |
| Optimism correction coefficient for R^2^ | 0.0628 | 0.0628 | 0.0408 | **0.0373** | 0.0106 | 0.0466 |
| Optimism correction coefficient for adj. R^2^ | 0.0663 | 0.0663 | 0.0422 | **0.0384** | 0.0107 | 0.0484 |
| Optimism-corrected R^2^ | 0.2907 | 0.2907 | 0.3035 | **0.3021** | 0.2749 | 0.2990 |
| Optimism-corrected adj. R^2^ | 0.2511 | 0.2511 | 0.2794 | **0.2808** | 0.2667 | 0.2719 |

Data are expressed as β values ± standard errors. HDL-c: high-density lipoprotein cholesterol; METs: metabolic equivalents; BMI: body mass index; TFAT: total body fat; VFAT: visceral fat; GRS_HDL-c: genetic risk score for high-density lipoprotein cholesterol; LARS: least-angle regression; BSRP: best subset regression procedure; BSM: bootstrapping stepwise method; AIC: akaike information criterion; AICC: corrected akaike information criterion; BIC: bayesian information criterion. Values in bold indicate the best predictive model.

**Supplementary Table 3d.** Multiple linear regression models explaining triglyceride levels as dependent variable.

|  |  | BSRP | | |  |
| --- | --- | --- | --- | --- | --- |
| Predictors | LARS | Adj. R^2^ | AIC/AICC | BIC | **BSM** |
| Age (years) | 0.35±0.34 |  |  |  |  |
| Sex |  | 15.26±9.96 | 16.69±9.74 |  |  |
| Alcohol | -18.95±9.57 | -16.54±9.65 | -19.78±9.49 |  | **-19.21±9.33** |
| METs | -0.22±0.16 | -0.22±0.16 |  |  |  |
| Energy intake (100 kcal) | 0.56±0.34 | 0.58±0.34 | 0.57±0.34 |  |  |
| BMI (kg/m^2^) |  | 2.30±1.89 |  |  |  |
| TFAT (kg) | -1.12±0.44 | -2.34±0.82 | -1.52±0.47 |  | **-1.14±0.42** |
| VFAT (kg) | 28.50±4.39 | 33.24±5.46 | 35.74±5.16 | 26.04±3.62 | **30.95±3.92** |
| GRS_TG | 4.20±0.99 | 4.18±0.98 | 4.28±0.97 | 4.13±0.98 | **4.20±0.97** |
| Constant | 36.88±26.69 | 6.64±38.96 | 39.11±19.87 | 24.71±10.25 | **61.58±17.31** |
| R^2^ | 0.2955 | 0.3028 | 0.2986 | 0.2554 | **0.2828** |
| Adj. R^2^ | 0.2754 | 0.2800 | 0.2819 | 0.2496 | **0.2715** |
| Optimism correction coefficient for R^2^ | 0.0382 | 0.0413 | 0.0326 | 0.0112 | **0.0211** |
| Optimism correction coefficient for adj. R^2^ | 0.0392 | 0.0426 | 0.0333 | 0.0113 | **0.0214** |
| Optimism-corrected R^2^ | 0.2573 | 0.2615 | 0.2660 | 0.2442 | **0.2617** |
| Optimism-corrected adj. R^2^ | 0.2362 | 0.2374 | 0.2486 | 0.2383 | **0.2501** |

Data are expressed as β values ± standard errors. TG: triglycerides; METs: metabolic equivalents; BMI: body mass index; TFAT: total body fat; VFAT: visceral fat; GRS_TG: genetic risk score for triglycerides; LARS: least-angle regression; BSRP: best subset regression procedure; BSM: bootstrapping stepwise method; AIC: akaike information criterion; AICC: corrected akaike information criterion; BIC: bayesian information criterion. Values in bold indicate the best predictive model.
